# ClereMed: Lessons Learned From a Pilot Study of a Mobile Screening Tool to Identify and Support Adults Who Have Difficulty With Medication Labels

**DOI:** 10.2196/mhealth.3250

**Published:** 2014-08-15

**Authors:** Kelly Anne Grindrod, Allison Gates, Lisa Dolovich, Roderick Slavcev, Rob Drimmie, Behzad Aghaei, Calvin Poon, Shamrozé Khan, Susan J Leat

**Affiliations:** ^1^School of PharmacyUniversity of WaterlooWaterloo, ONCanada; ^2^School of Public Health and Health SystemsUniversity of WaterlooWaterloo, ONCanada; ^3^Department of Family MedicineMcMaster UniversityHamilton, ONCanada; ^4^CommunitechKitchener, ONCanada; ^5^School of Optometry and Vision ScienceUniversity of WaterlooWaterloo, ONCanada

**Keywords:** low vision, legibility, prescription labelling, medication labels, usability, cognitive impairment, visual impairment

## Abstract

**Background:**

In order to take medications safely and effectively, individuals need to be able to see, read, and understand the medication labels. However, one-half of medication labels are currently misunderstood, often because of low literacy, low vision, and cognitive impairment. We sought to design a mobile tool termed ClereMed that could rapidly screen for adults who have difficulty reading or understanding their medication labels.

**Objective:**

The aim of this study was to build the ClereMed prototype; to determine the usability of the prototype with adults 55 and over; to assess its accuracy for identifying adults with low-functional reading ability, poor ability on a real-life pill-sorting task, and low cognition; and to assess the acceptability of a touchscreen device with older adults with age-related changes to vision and cognition.

**Methods:**

This pilot study enrolled adults (≥55 years) who were recruited through pharmacies, retirement residences, and a low-vision optometry clinic. ClereMed is a hypertext markup language (HTML)-5 prototype app that simulates medication taking using an iPad, and also provides information on how to improve the accessibility of prescription labels. A paper-based questionnaire included questions on participant demographics, computer literacy, and the Systems Usability Scale (SUS). Cognition was assessed using the Montreal Cognitive Assessment tool, and functional reading ability was measured using the MNRead Acuity Chart. Simulation results were compared with a real-life, medication-taking exercise using prescription vials, tablets, and pillboxes.

**Results:**

The 47 participants had a mean age of 76 (SD 11) years and 60% (28/47) were female. Of the participants, 32% (15/47) did not own a computer or touchscreen device. The mean SUS score was 76/100. ClereMed correctly identified 72% (5/7) of participants with functional reading difficulty, and 63% (5/8) who failed a real-life pill-sorting task, but only 21% (6/28) of participants with cognitive impairment. Participants who owned a computer or touchscreen completed ClereMed in a mean time of 26 (SD 16) seconds, compared with 52 (SD 34) seconds for those who do not own a device (*P*<.001). Those who had difficulty, struggled with screen glare, button activation, and the “drag and drop” function.

**Conclusions:**

ClereMed was well accepted by older participants, but it was only moderately accurate for reading ability and not for mild cognitive impairment. Future versions may be most useful as part of a larger medication assessment or as a tool to help family members and caregivers identify individuals with impaired functional reading ability. Future research is needed to improve the sensitivity for measuring cognitive impairment and on the feasibility of implementing a mobile app into pharmacy workflow.

## Introduction

### Background

The Institute of Medicine (IoM) estimates that each year at least 1.5 million deaths in the United States are caused by preventable adverse drug events with patient confusion as a major contributor [1-6]. To save lives, the IoM and others, including the Institute for Safe Medication Practices (ISMP) and the American Foundation for the Blind, have urged health professionals to communicate more clearly, improve the legibility of medication labels, and provide information in ways that are accessible to adults with low vision and low literacy [2,7,8]. Patients are also being encouraged to take a more active role by maintaining careful records and double checking their own prescriptions [2].

Yet, to use, record, double check, and understand a prescription, a patient or caregiver must be able to see, read, and understand their medication labels. An estimated 1 in 12 North American adults have a self-reported “seeing disability,” with eye diseases, such as cataracts, glaucoma, diabetic retinopathy, or macular degeneration being major causes of vision loss [9-11]. In a study of simulated vision loss, 3-point font is at the limit of legibility for healthy vision, whereas mild- to moderate-simulated vision loss renders anything smaller than 8 to 14 points illegible [12]. The result is that regular prescription medication labels cannot be read accurately by those with moderate-simulated visual impairment. Many older adults also have difficulties reading if there is glare or dim lighting, and struggle with low-contrast reading materials [13].

Individuals over age 65 who are visually impaired are 2 to 3 times more likely to need help managing their medications [14-16]. Specifically, older adults have more difficulty recalling and understanding information printed on prescription labels in 7-point font than in 10-point font [17]. Older adults also read labels less accurately when printed material is in 9- or 12-point font rather than 14-point font [18]. However, Leat et al [19] found that while reading speed is slower for participants with visual impairment, accuracy of reading is high if participants are allowed to use their magnification devices, suggesting that simple interventions can improve prescription label readability.

Patients who can see medication labels must also be able to read and understand the presented information. Just under one-half of North American adults have low literacy (47% in the United States and 48% in Canada) meaning they lack the literacy skills needed for everyday life [20,21]. Between 46% and 60% also have low health literacy and struggle to “obtain, process, and understand basic health information and services needed to make appropriate health decisions” [22,23].

In addition, 1 in 5 community-dwelling adults aged 65 and older have cognitive impairment, including 5-7% who have dementia [24-26]. Compared with individuals with healthy cognition, those with cognitive impairment are 2 to 4 times more likely to be nonadherent to their medication therapy [27,28]. Because 1 in 4 home-dwelling adults with cognitive impairment also have vision problems [29], many patients have multiple barriers to taking medications safely and effectively.

### Identifying the Problem

Considering that one-half of all prescription labels are misunderstood, there is a significant need to identify individuals who require additional support with their medication therapy [6]. For chronic conditions such as diabetes, cardiovascular disease, and mental illness, at least one-half of patients are nonadherent to prescribed therapy often because they have a poor understanding of the purpose, safety, and effect of the medications they have been prescribed [30-34].

Despite the high prevalence of medication misunderstandings, adverse events, and nonadherence, there is no gold standard screening tool that health professionals can use at the point-of-care to identify people who need additional support to use medications safely and effectively. In fact, results from Leat’s lab show that 94% of participants (older adults and adults with low vision) reported not being asked by their pharmacist whether they had difficulty reading labels, while 90% of pharmacists reported that there were either no guidelines or they did not know if there were guidelines on when to ask patients if they would like large print (Susan J Leat, PhD, email communication, June 22, 2014).

In research studies of patient comprehension and adherence, the most common screening strategies have been to ask patients to use standard dosing instructions to either calculate the total daily dose being prescribed or to fill a pillbox [35-37]. In a recent study, Anderson et al [38] compared both methods in 65 multi-ethnic patients with type 2 diabetes and found that the pillbox fill was a more conservative method for identifying patients with low cognition [38]. Another strategy that has been proposed is for pharmacists to assess health literacy using the Very Short Test of Functional Health Literacy in Adults, which is positively correlated with pharmacy comprehension (*r*=.72, *P*≤.001) [39]. However, neither of these strategies have been widely adopted in front-line pharmacies.

### Making Medication Information More Accessible

One of the first steps to making medication information more accessible is to identify individuals who simply cannot see the medication information or instructions. On a typical pharmacy label, the most prominent and legible information is the pharmacy logo, which has a mean font size of 13.6 points [40]. By comparison, medication instructions, medication names, and warning labels are 9.3, 8.9, and 6.5 points, respectively [40]. In a recent study of Canadian prescription labels, 44% of medication instructions and all drug and patient names were smaller than 12-point font [41].

To improve legibility, the United States Pharmacopeia recommends that pharmacists label prescriptions with a high contrast print, a simple uncondensed large font (eg, 11-point font, Arial), and adequate white space [42]. Health Canada also recommends pharmacists use a minimum 10-point font [43]. More specific to patients with low vision, the American Foundation for the Blind, the American Society of Consultant Pharmacists Foundation, and ISMP have recommended that pharmacists give patients the option of having large print prescription labels in at least 18-point font [7,8].

In recent years, there has also been considerable effort put toward creating accessible health information. Health Canada has begun producing plain language medication handouts that are more accessible to patients [44]. In the United States, the National Patient Safety Foundation is promoting the “Ask Me 3” campaign that encourages patients to improve their health literacy by preparing questions ahead of physician visits [45]. The Agency for Healthcare Research and Quality has also developed an in depth pharmacy health literacy assessment process to better understand health literacy by guiding pharmacies in collecting information from patients, staff, and objective auditors through a series of surveys and focus groups [46].

### The ClereMed Mobile App

The purpose of the study was to build a mobile tool that could be easily used by health care providers or caregivers to identify individuals who have difficulty reading or understanding medication labels and to study the accuracy, usability, and acceptability of the device. To guide content, development, and usability, we convened an advisory committee including a pharmacist and pharmacy professor (Kelly Grindrod); a representative from the Canadian National Institute for the Blind (Deborah Gold); an optometry professor and researcher in the area of low vision, visual assessment, and reading (Susan Leat); an optometrist working with low vision patients (Shamroze Kahn); a pharmacy business expert (Roderick Slavcev); a community pharmacist (Bryan Hastie); and a pharmacy student with a special interest in eHealth technologies (Calvin Poon). The committee met monthly until the final prototype was developed. This approach is consistent with the third generation participatory design framework for health informatics development [47].

As described above, the overall goal was to study one approach to help pharmacists identify patients over age 55 who have difficulty reading and/or understanding the instructions on prescription medications. We designed the app by focusing on the users, the task, and the outcome.

We began by writing use-cases for patients we encountered who had struggled to read prescription labels. We defined our patient users as individuals over age 55 who use at least one chronic medication, including narrow therapeutic index drugs, such as warfarin and insulin. In describing our users, we made several assumptions. First, we assumed some individuals over age 55 may not be familiar with computers and may require extra support to use a touchscreen device. We also noted some individuals with functional impairments find it difficult to complete paper questionnaires and find touchscreen devices more user-friendly [48]. Given that we were hoping to influence the legibility of prescription labels, we also assumed that it would be most effective to have the pharmacist administer the screening test to the patient rather than having the patient complete the task at home. Our rationale was that the pharmacist could screen the patient when they filled a new prescription and immediately apply the findings to prescription labeling.

We originally intended to design an app that could rapidly screen patients for visual impairments. With the use-cases, we put together several ideas including questionnaires, vision screening tests, and simulations. In consultation with the advisory committee, we chose to develop an app to simulate medication taking and hypothesized it would also capture individuals who could see a label, but could not understand the instructions due to low literacy or cognitive impairment. Although a paper screener or an actual pill vial may have some advantages (simplicity, less expense), an electronic version has the advantage that the results could potentially be stored in the patient’s e-record, be shared with other health care providers, automatically print large-print labels, or generate a recommendation for an eye examination.

**Figure 1:**
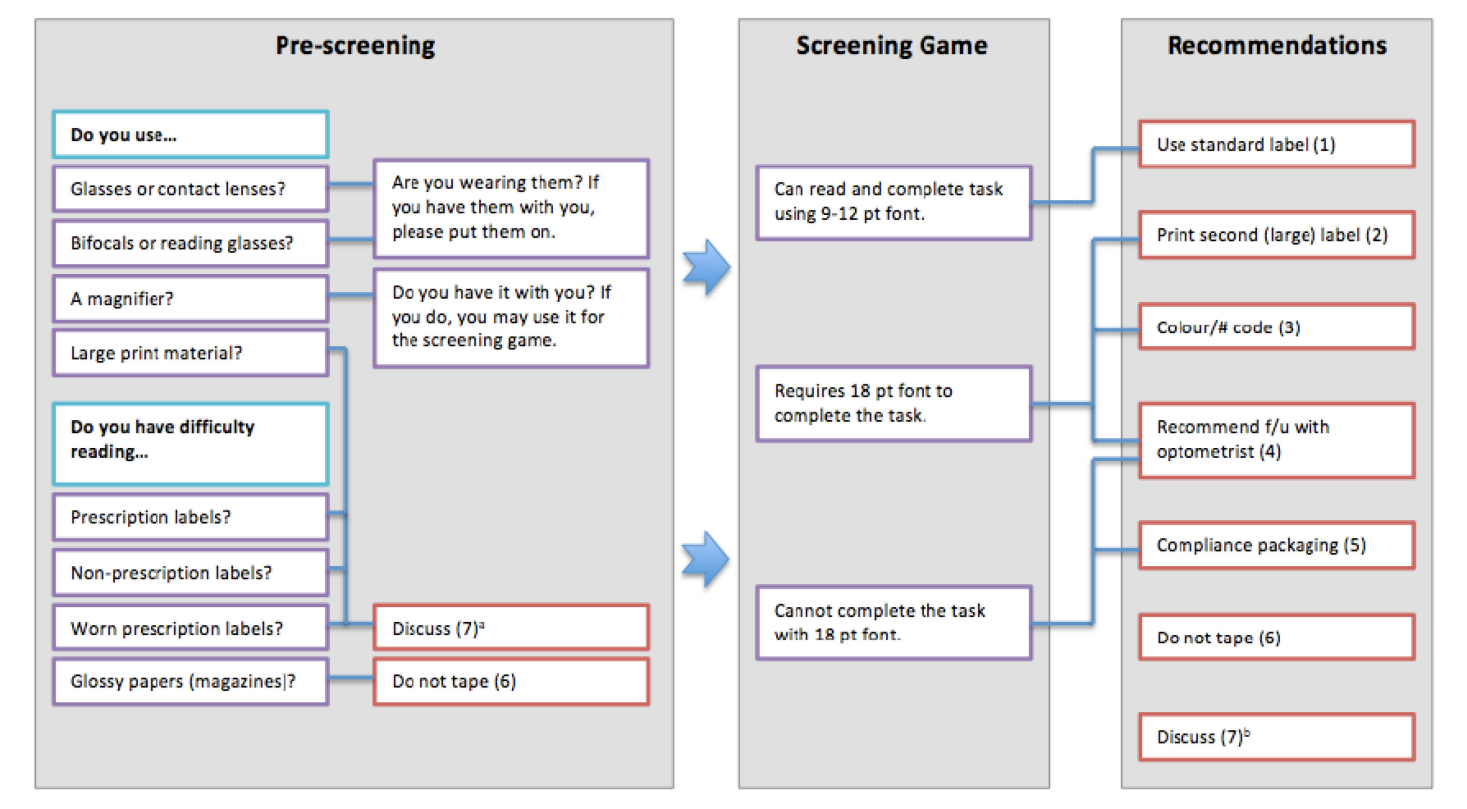
Flow chart linking ClereMed prescreening and screening test results to recommendations. (a): Although a participant may report requiring large print material or having difficulty reading prescription or OTC labels and/or worn labels, screening results may indicate that the participant has the ability to complete the screen game using 9-12pt font. In this case, the pharmacist should discuss the results with the participant and decide on the best option. (b): When applicable based on screening results, prescription(s), or disease condition(s) (eg, if participant reports difficulty with both worn and glossy labels, the benefits and risks of taping the label should be discussed).

The researchers worked with a systems design trainee (Behzad Aghaei) to mock-up the app using the Balsamiq platform. To maximize usability, we highlighted the importance of large fonts, large buttons, and intuitive colors (eg, green buttons to move to the next screen). In addition, to help pharmacists act on the results of the screening tool, we developed a prescription-labeling algorithm using the recommendations from the American Foundation for the Blind and the American Society of Consultant Pharmacists Foundation (Figure 1) [7].

The app prototype was built through the Communitech Apps Factory in Kitchener ON, by co-op students from the University of Waterloo computer science program. The app was programmed using hypertext markup language (HTML)-5 and designed for the Apple mobile Operating System (iOS) [49].

**Figure 2:**
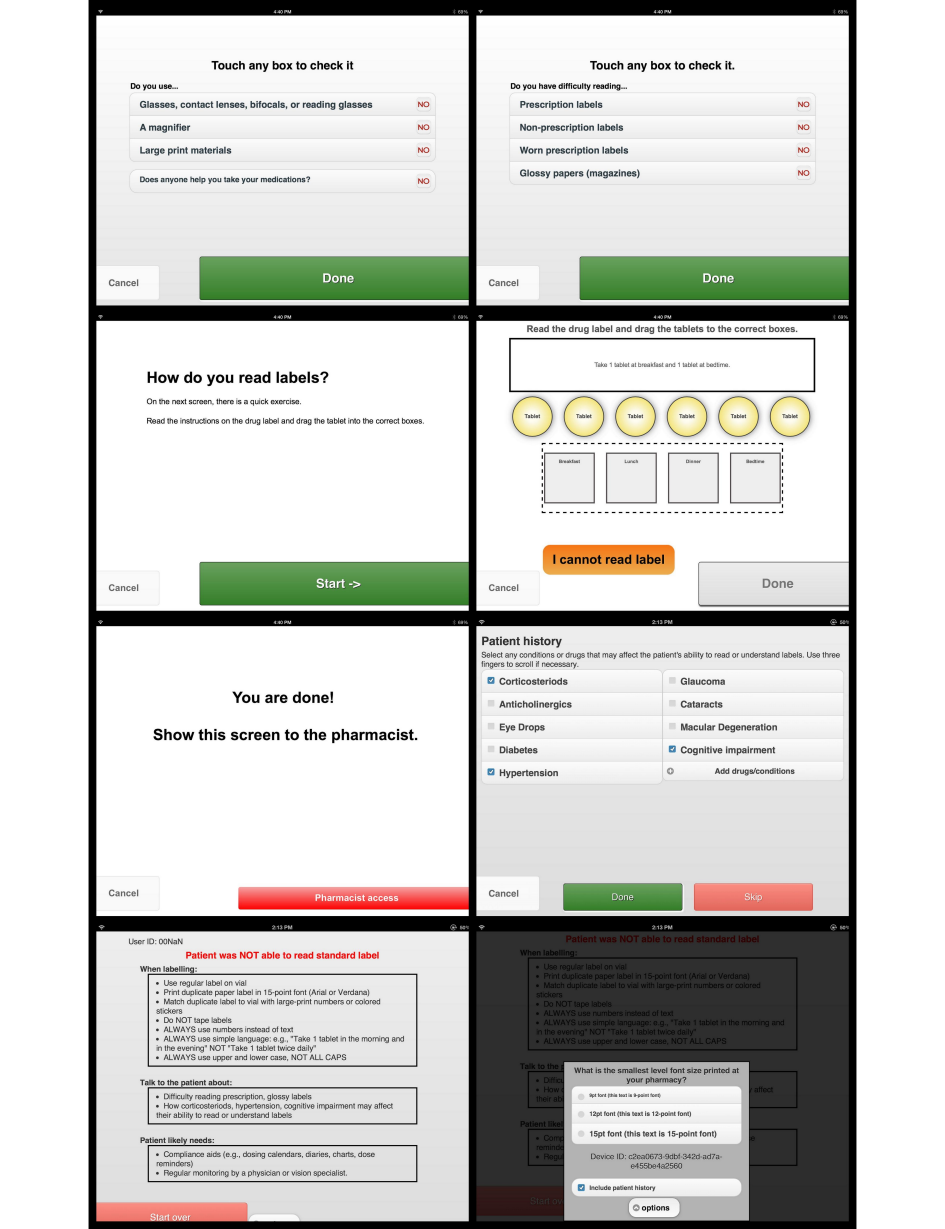
Screenshots of ClereMed.

We termed the app “ClereMed” (Figure 2). The final prototype included two phases and was designed to take 2 minutes to complete. During the first phase (patient-directed), patients were asked about their perceived ability to read prescription and nonprescription labels. Patients then completed a 1-minute simulation where they read a hypothetical prescription label written in 6.5-point font and followed the instructions to correctly drag and drop “tablets” into the correct times on a “pillbox”. If the patient could not accurately and easily complete the task, the patient was prompted to repeat the activity using progressively larger font sizes. Participants were allowed to increase the print until they felt they could undertake the task comfortably. Of interest, on analysis we identified that, despite programming ClereMed to use default font sizes of 9, 12, 15, and 18 points, the font-sizes in the actual app appeared as 6.5, 9, 11, and 13 points, respectively. The “drawing and print guide for iOS” confirms that font sizes are device-dependent [50].

In the second phase (pharmacist-directed), the pharmacist was asked to identify any common medication-related issues that could reduce visual acuity (the smallest detail that an eye can discern), such as uncontrolled diabetes or chronic corticosteroid use. A participant was considered to have failed the visual aspect of the app if they required the print to be larger than the second largest print size on the app (ie, >9 points in actual size). This cut point was chosen as then a patient would be able to read most patient-critical information on current prescription medication labels. A cut point of 11 points would mean that the majority of information would not be legible [40]. The app closed with patient-specific recommendations to improve prescription label legibility based on the outcomes of the screening questions and simulation.

### Research Question and Objectives

Our research question asks, can an easy-to-use mobile app be designed to help pharmacists identify and support adult patients over age 55 who may have difficulty reading or understanding prescription labeling? The goal of the app was to provide patients and pharmacists with realistic and individualized recommendations to improve the legibility of labels before their patients leave the pharmacy and to assess their ability to follow instructions.

The specific objectives of this pilot study were to build the ClereMed prototype, to determine the usability of the prototype with adults 55 and over, and to assess its accuracy for identifying adults with low-functional reading ability, poor ability on a real-life pill-sorting task, and low cognition. We also wished to assess the acceptability of a touchscreen device with older adults with age-related changes to vision and cognition and to provide recommendations for future mobile app development.

## Methods

### Participants

Our methods and results are reported according to the CONSORT-EHEALTH statement [51]. This study was reviewed and received ethics clearance through the Office of Research Ethics at the University of Waterloo on January 6, 2012 (Application #17596).

We pilot-tested ClereMed with individuals age 55 and over, who could speak and read English and who were taking at least one chronic medication. We recruited participants from the Waterloo Region, which is a large urban center in Southwestern Ontario with a population of over 500,000.

We began our pilot testing by asking pharmacists to test the app in their pharmacy. We asked pharmacists to approach patients who were filling a prescription and ask if they would be willing to test a new mobile app. After very poor initial uptake, we moved the pilot testing out of the pharmacy and into local retirement residences and a low-vision clinic at the University of Waterloo School of Optometry and Vision Science. Results for the pharmacist directed portion of the ClereMed will not be described due to low uptake of ClereMed by pharmacists. Potential participants were contacted by telephone, mail, and using posters and were invited to schedule a time to meet with a research assistant. They were given the option to test ClereMed at their home, in the common areas of their retirement home (if applicable), or in the clinical spaces of the University of Waterloo Schools of Pharmacy, Optometry, and Vision Science. All participants provided written consent prior to taking part in the study and were provided with a $10 gift certificate in appreciation of their time.

Halfway through pilot testing we made minor updates to the app. Participants were originally required to tap radio buttons. In response to user difficulty with the tapping action, we changed radio buttons to yes/no “sliders”. We also enlarged the button sizes and participants were offered the choice of using a stylus.

### Data Collection

Immediately before trying ClereMed, the research assistant asked participants to complete a paper-based background questionnaire that included questions on patient demographics and computer experience. Participants tested ClereMed on an iPad using any assistive devices they would typically use to read a prescription label (eg, spectacles, a magnifying glass). The research assistant provided little guidance and only offered a prompt if the participant could not move forward with a task after several attempts.

After completing the app, participants assessed usability with the Systems Usability Scale (SUS) and by providing written feedback [52]. The SUS is a validated tool that uses a 5-point Likert scale to provide a quantitative measure of the usability and learnability of a system and provides an overall score between 0 and 100 [52]. A trained research assistant administered a vision and cognitive assessment. We assessed reading visual acuity using the MNRead Near Vision Chart. Participants used the same spectacles as were used for the app and held the chart at 40 cm with good, even illumination. To achieve the clearest vision for the testing, +2.00 lenses were held over the participant’s habitual distance prescription or in a trial frame if this provided clearer vision. Functional visual impairment was designated as visual acuity >1 M on the MNread chart (equivalent to Arial >8.5-point font, which is similar to the cut off used for the app).

We assessed cognition using the Montreal Cognitive Assessment tool (MoCA) [53]. The MoCA is a validated, paper-based test that is used to detect mild cognitive impairment. It has short questions that test the areas of visuospatial, naming, memory, attention, language, abstraction, delayed recall, and orientation. It was chosen because it has a greater sensitivity for detecting mild cognitive impairment compared with the Mini-Mental State Examination [53]. MoCA scores of ≤25/30 were defined as mild cognitive impairment.

Lastly, as a true-life task, participants completed a real-life prescription vial simulation. Participants were handed a pill bottle with instructions written in Arial, 9-point font (eg, “Take ONE tablet THREE times daily”) and asked to place the pills into a pillbox in accordance with the instructions. Participants who could not complete the activity were given pill bottles labeled with incrementally larger font sizes and asked to try again, until they were able to do the task correctly. A research assistant recorded the time taken to complete the activity using a digital timer.

### Statistical Analysis

Data were analyzed using SPSS. We used descriptive statistics to summarize demographic data. For each factor of the SUS, participant responses to the 5-item Likert agreement scale were scored by subtracting 1 from odd numbered factors, subtracting the even numbered factors from 5 and multiplying each factor by 2.5 for a total possible score of 100 for the entire 10-item scale [54]. The total mean usability score as well as the scores for learnability (factors 4 and 10) and usability (factors 1-3 and 5-9) [55] were compared between the populations of participants who did and did not own a computer/touchscreen device using a Mann-Whitney test for nonparametric data.

We converted the MNRead scores (which are in a Times Roman font) to the Arial font size equivalent. Both MNRead scores and app print size were log transformed, as is usual for vision data. The correlation between the near-visual acuity score and the smallest font size required by the participant to complete the app was calculated using a Pearson product-moment correlation coefficient. We assessed agreement using a Bland-Altman plot. To determine the accuracy of ClereMed, participants’ ability to complete the app without help was compared against the MNRead, real-life pill bottles, and MoCA results (sensitivity and specificity). Statistical significance for all tests was determined a priori at a level of *P*<.05.

## Results

### Participants

Over a 2-month period, a total of 4 participants were recruited through two pharmacies. The study protocol was expanded to nonpharmacy environments and within 2 months, 39 participants were recruited from retirement residences and 4 additional participants were recruited from the low-vision clinic.

Of the 47 participants who completed the study, 60% (28/47) were female, the mean age was 76 (55-93 years), 15% (7/47) had functional visual impairment based on the MNread visual acuity, and 62% (29/47) had mild cognitive impairment based on the MoCA (Table 1). Of participants, 77% (35/47) reported having at least one condition that could affect ability to see and/or understand prescription labels. Further, 32% (15/47) of participants did not use a computer, tablet, e-reader, or mobile phone at home. Participants who had access to a computer or touchscreen device at home completed ClereMed in a mean time of 26 (SD 16) seconds, compared with 52 (SD 34) seconds for those who did not (*P*=.001).

In their daily life, nearly all participants wore spectacles, while 21% (10/47) used a magnifier and 21% (10/47) used large print materials. Of the participants, 14% (6/44) needed assistance to take their medications and 36% (16/44) reported having difficulty reading medication labels or nonprescription labels. Worn or glossy labels were also a problem for 39% (17/44). The most common complaints about the legibility of prescription labels were that the fonts were too small and that the contrast was poor. Many participants reported that labels are easier to read when large letters, bold fonts, and high contrast were used.

**Table 1:** Participant demographics, technology in the home, and self-reported difficulty reading medication labels (N=47).

Demographics		N	%
Age, years, mean (range)		76 (55-93)	
Female		28	60
**Highest education level completed**			
	High school	20	43
	Trade school	1	2
	Post-secondary	21	45
	Graduate degree	5	10
**Annual income ($CAD)**			
	<20,000	7	15
	20,000-49,000	15	32
	50,000-79,999	4	9
	>80,000	4	9
	Prefer not to respond	17	36
**Technology in the home**			
	Computer	30	63
	Tablet	4	9
	e-Reader	10	21
	Mobile phone	4	9
**Assistive devices for daily living**			
	Spectacles	42	89
	Magnifier	10	21
	Large print	10	21
**Self-reported difficulty**			
	Taking medications	6	13
	Reading prescription labels	12	26
	Reading nonprescription labels	14	30
	Reading worn labels	15	32
	Reading glossy paper	9	19
	Concerned about ability to read or understand medication labels	10	21
**Medical conditions that could affect ability to read medication labels**			
	Diabetes	7	15
	Hypertension	13	28
	Glaucoma	6	13
	Cataracts	12	26
	Macular degeneration	4	9
	Total with at least one condition affecting vision	35	77
**Medications that could affect ability to read medication labels**			
	Corticosteroids	0	0
	Anticholinergics	1	2
	Medicated eye drops	13	27
	Cognitive impairment (MoCA <25/30)	29	62
	Functional visual impairment (MNRead <1M)	7	15

### ClereMed Usability

Overall, the mean SUS was 76/100 (Table 2), with 84% (37/44) of participants agreeing the app was easy to use on item 3. Participants who owned computers or touchscreen devices found ClereMed to be more usable than those who did not own a computer or touchscreen (mean SUS score difference 16.22, 95% CI 11.59-20.86, *P*<.03; Table 3).

The difference may be largely attributable to the learnability of ClereMed (items 4 and 10), with 60% (9/15) of those with no technology ownership agreeing or strongly agreeing they would require some kind of technical support to get through it (SUS Factor 4) and 47% (7/15) agreeing or strongly agreeing they needed to learn a lot before using ClereMed (SUS Factor 10), compared with a respective 24% (7/29) and 17% (5/29) in the technology ownership group.

In written feedback, most participants found the app to be simple and thought it could quickly identify patients with visual impairment within a pharmacy. Some positive comments included that the screen was a nice size, the app had good contrast, instructions for the simulation activity were clear, and the process was quick and concise. Of participants who found the app difficult to use, many had trouble navigating the touchscreen, either due to lack of dexterity, hand tremor, or simply because the screen did not respond to their touch. This problem was often alleviated with the use of a stylus.

Some participants noted that the font sizes for the simulation instructions were too small and felt larger, bolder fonts would make it easier to complete the app. Many participants also had trouble with screen glare. Of the visually impaired participants, some noted that the yellow color used for the tablets in the simulation was hard to see. They also noted that white fonts on a black background might be easier to read. Finally, some participants noted that radio buttons (for yes/no responses) were confusing, while others were not familiar with the term “drag” for the drag and drop simulation.

With regards to the minor updates made to the app during the pilot, the larger buttons did appear to reduce user difficulties but the change from radio buttons to a yes/no slider did not.

**Table 2:** Participant agreement with Systems Usability Scale (SUS) items after using ClereMed (1=strongly disagree, 2=disagree, 3=neutral, 4=agree, 5=strongly agree) and mean SUS score (n=44^a^).

	Mean Agreement (SD)	MeanSUS Score (SD)^b^
1. I think that I would like to use ClereMed frequently	3.05 (1.71)	5.11 (4.28)
2. I found ClereMed unnecessarily complex	1.70 (1.42)	8.24 (3.56)
3. I thought ClereMed was easy to use	4.39 (1.35)	8.47 (3.38)
4. I think that I would need the support of a technical person to be able to use ClereMed	2.52 (1.59)	6.19 (3.98)
5. I found the various functions in ClereMed were well integrated	4.25 (1.24)	8.13 (3.10)
6. I thought there was too much inconsistency in ClereMed	1.45 (1.04)	8.86 (2.61)
7. I would imagine that most people would learn to use ClereMed very quickly	4.14 (1.37)	7.84 (3.44)
8. I found ClereMed very cumbersome to use	1.55 (1.23)	8.64 (3.07)
9. I felt very confident using ClereMed	4.16 (1.29)	7.90 (3.23)
10. I needed to learn a lot of things before I could get going with ClereMed	2.27 (1.72)	6.82 (4.29)
Learnability score^c^	-	65.06 (35.72)
Usability score^d^	-	78.98 (20.19)
Total SUS score	-	76.19 (20.67)

^a^Three participants who had severe vision impairment tried but could not test the app.

^b^Odd numbered items (1, 3, 5, 7, 9) were scored by subtracting 1 from the mean agreement and multiplying by a factor of 2.5. Even numbered items (2, 4, 6, 8, 10) were scored by subtracting the mean agreement from 5 and then multiplying by a factor of 2.5.^51^

^c^Learnability is represented by factors 4 and 10.

^d^Usability is represented by factors 1-3 and 5-9.

**Table 3:** Responses to Systems Usability Scale (SUS) components according to computer and touchscreen ownership for participants who could use ClereMed (n=44^a^).

Responses	Mean SUS score (SD)	
	Technology ownership (n=29)	No technology ownership (n=15)
1. I think that I would like to use ClereMed frequently^b^	6.55 (3.68)	2.33 (4.06)
2. I found ClereMed unnecessarily complex^b^	9.22 (2.23)	6.33 (4.81)
3. I thought ClereMed was easy to use	9.05 (2.45)	7.33 (4.58)
4. I think that I would need the support of a technical person to be able to use ClereMed	7.07 (3.60)	4.50 (4.25)
5. I found the various functions in ClereMed were well integrated	7.59 (3.57)	9.17 (1.54)
6. I thought there was too much inconsistency in ClereMed	9.14 (2.03)	8.33 (3.49)
7. I would imagine that most people would learn to use ClereMed very quickly	7.76 (3.36)	8.00 (3.68)
8. I found ClereMed very cumbersome to use	8.79 (2.80)	8.33 (3.62)
9. I felt very confident using ClereMed^b^	8.79 (2.07)	6.17 (4.32)
10. I needed to learn a lot of things before I could get going with ClereMed	7.76 (3.68)	5.00 (4.91)
Learnability score^b^ (*P*=.04)	74.14 (28.53)	47.50 (42.31)
Usability score^c^ (*P*=.06)	83.62 (16.32)	70.00 (24.26)
Total SUS score (*P*=.03)	81.72 (15.78)	65.5 (25.06)

^a^Three participants who had severe vision impairment tried by could not test the app.

^b^Odd numbered items (1, 3, 5, 7, 9) were scored by subtracting 1 from the mean agreement and multiplying by a factor of 2.5. Even numbered items (2, 4, 6, 8, 10) were scored by subtracting the mean agreement from 5 and then multiplying by a factor of 2.5.^51^

^c^Learnability is represented by factors 4 and 10.

^d^Usability is represented by factors 1-3 and 5-9.

### Accuracy

In terms of vision, ClereMed correctly identified 71% (5/7, sensitivity) of participants who had functional vision impairment and 86% (31/36, specificity) who had healthy, functional vision (Table 4). There was a positive correlation between the log MNRead visual acuity and the log smallest app print size read (*r*=.56, n=45, *P*<.001). However, this was strongly influenced by one outlier who had extreme low vision (MNread reading acuity value of 1.5), and when this was removed the correlation fell to *r*=.43 (n=45, *P*=.003). Of particular note, it was not apparent that the individual had such poor vision until they were asked to complete ClereMed and indicated they could not see the iPad.

**Table 4:** Sensitivity and specificity of ClereMed for identifying individuals with functional vision impairment, mild cognitive impairment, and who failed the real-life simulation (N=43^a^).

	Functional vision impairment	Mild cognitive impairment	Failed real-life simulation
Sensitivity	0.71 (5/7)	0.21 (6/28)	0.63 (5/8)
Specificity	0.86 (31/36)	1.00 (15/15)	0.97 (34/35)

^a^Due to a system error, results data was not collected for the first four participants who tested ClereMed.

**Figure 3:**
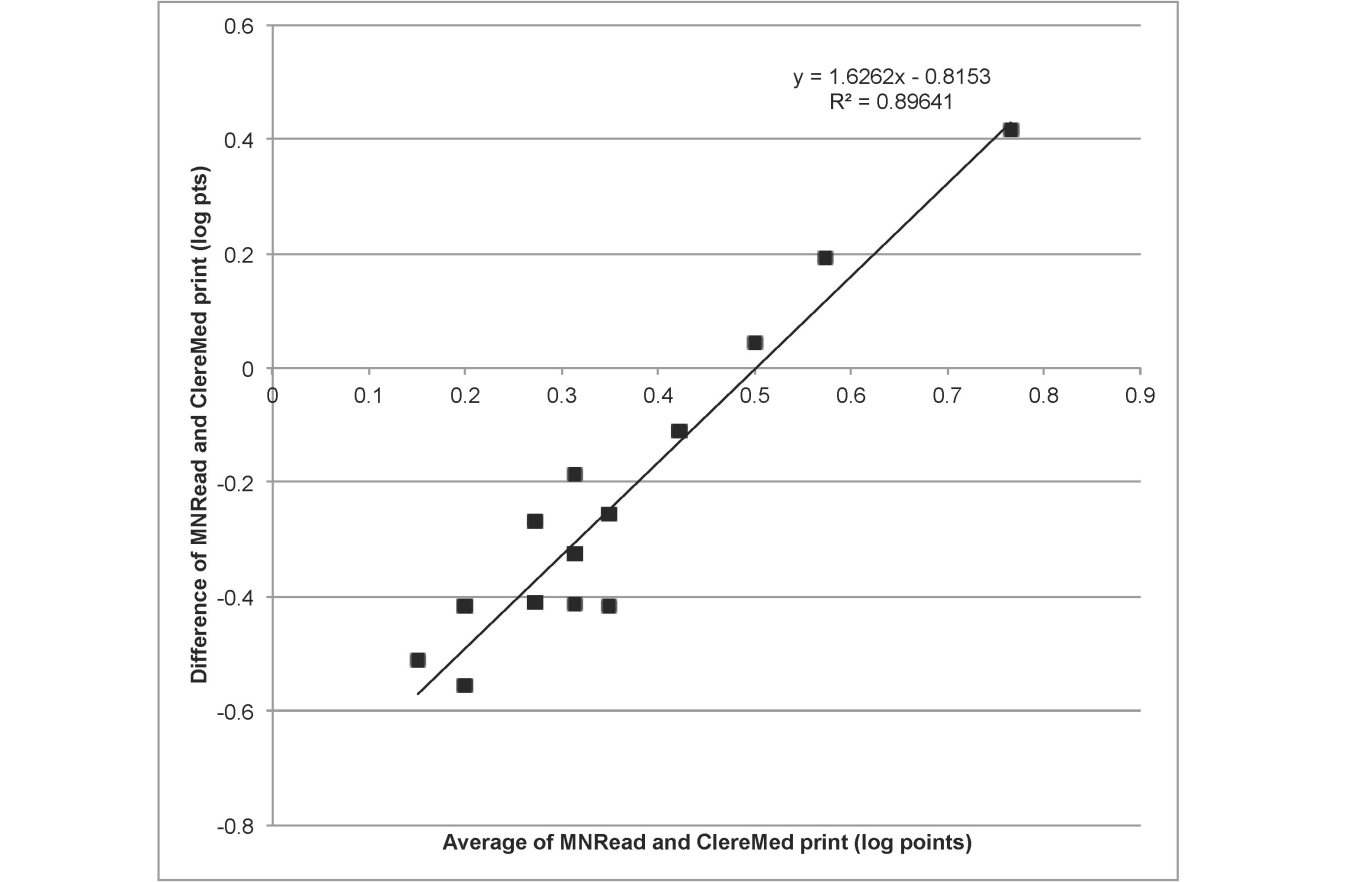
Bland Altman plot comparing MNRead near visual acuity results to the results of the ClereMed vision screening test (N=43).

The Bland-Altman analysis (Figure 3) showed that there was moderate agreement between the measures in terms of reading print. On average, the point print chosen for the app tended to be larger than the reading acuity measured with the MNRead. However, there was a strong trend toward those with better vision choosing a larger print on the ClereMed, while 2 participants with poorer vision chose smaller print on the app.

Compared with the real-life prescription vial simulation, ClereMed correctly identified 63% (5/8) of people who could not complete the simulation and 97% (34/35) of participants who could complete it (Table 4).

Of the 62% (29/47) of participants identified by the MoCA as having cognitive impairment, 2 individuals could not complete the ClereMed tool. One participant with healthy cognitive ability also had difficulty comprehending the instructions for the simulation activity. ClereMed correctly identified only 21% (6/28) participants with mild cognitive impairment but identified 100% (15/15) of participants who had healthy cognition.

## Discussion

### Principal Findings

ClereMed was moderately accurate for identifying participants who could not read prescription labels compared with the MNRead reading test and the real-life prescription vial simulation. It was not accurate for detecting mild cognitive impairment. Given ClereMed’s reported ease-of-use for adults 55 and over, the app may be a convenient option to estimate a patient’s ability to read a medication label. Although visual acuity can be measured simply on a reading card, the app has the advantage of not getting soiled or losing its contrast and can measure a variety of functions in addition to visual acuity. However, further development and testing of ClereMed is necessary before it could be integrated into the workflow of pharmacies and other health care settings.

Though there was moderately good correlation with reading acuity, most participants preferred print that was larger than their visual acuity threshold as measured by the MNRead. This is not surprising as the MNread measured the participant’s acuity limit, while ClereMed allowed participants to increase the print until they felt they could undertake the task comfortably. It is known that to obtain reasonable fluency in reading, the print needs to be at least twice as large as the acuity threshold [56] and patients prefer print that is somewhat larger than their acuity limit. The 2 participants who preferred smaller print on ClereMed may have been using a spectacle with a strong reading addition or holding the ClereMed closer than when their near reading acuity was measured with the MNRead.

Though the sensitivity of ClereMed was high for functional vision impairment, the sensitivity was very low for mild cognitive impairment. ClereMed was not designed to measure cognition directly, but the practical ability to understand and follow instructions. This finding is consistent with a small study by Anderson et al [57] who, in 2008, found that the well-validated Mini-Mental State Exam for cognition was poorly correlated with the patient’s ability to fill a pillbox (*r* ^2^=.15, *P*=.046). Stilley et al [58] has also shown attention/psychomotor speed are cognitive domains that most consistently predict medication adherence. Executive function is also considered to be a good predictor of everyday functioning and includes behaviors such as purposive action/self-regulation, planning/attention, volition/inhibition, and effective performance/self-monitoring [59]. In 2013, Zartman et al [60] developed the “Pillbox Test” to assess executive function. Using five pill bottles with different colored beads, patients were asked to fill a pillbox using a range of common instructions ranging from “take one tablet daily” to “take one tablet in the am and pm” and “take one tablet every other day” [60]. Initial testing with 120 patients showed that the Pillbox Test was well correlated with the Direction Assessment of Functional Status score for executive function and that it had a sensitivity of 75% and a specificity of 87.5% for adults with neurological disorders including dementia and stroke compared with healthy adults [60]. For assessing cognition, future research on tests such as ClereMed should consider the role of the different cognitive domains.

### ClereMed Usability

In terms of usability, interacting with new technology at an older age without any previous experience can, understandably, be daunting. Many of the participants had previous computer experience, though much fewer had experience with touchscreen devices. The majority of participants, regardless of their previous experience with touchscreens, reacted positively to ClereMed. In general, participants found the app to be simple and easy to use and felt most people would learn to use it quickly. Given that many adults 55 and over would not have any previous experience using a touchscreen device, the reported usability of ClereMed is encouraging. Certainly, previous research has shown that many adults 55 and over, especially those with motor difficulties (eg, rheumatoid arthritis) may actually prefer using touchscreens over traditional pen and paper when completing questionnaires [48].

Many participants also expressed their apprehension prior to participating, stating their lack of experience as a reason to fear using an app. A review by Broady et al [61] reported that this is a typical reaction for adults 55 and over. Despite lack of experience that may lead them to feel less comfortable and competent using a new technology, personal relevance of the technology is an important factor in encouraging adults 55 and over to make use of such services [61]. In this study, most participants were pleasantly surprised at how quickly they picked up on the new technology. Knowing the relevance of the app to their everyday lives seemed to help participants want to learn how to use it.

Although most of the feedback provided about ClereMed was positive, some participants had difficulty interacting with the touchscreen. Many could not get the screen to respond to their touch and reported difficulty when finer movements were required (ie, when trying to tap a radio button). Most commonly, problems were encountered when there was a long lag time between touching the screen (pressing) and letting go (releasing). When this occurred, the system would not recognize the tap or would activate a copy/paste function until participants mastered the required technique. Leonardi et al [62] observed a similar problem when testing a touchscreen interface for older adults. They found that many misunderstood the tapping gesture. For some participants, up to a 1-second gap was measured between the “press” and the “release”. In other instances, the finger would move slightly while pressing, leading the system to interpret the motion as a dragging gesture, rather than a tap [62].

Research by Wacharamanotham et al [63] also found that elderly users with a hand tremor may have difficulty interacting with touchscreens due to finger oscillation. Instead of tapping, a “swabbing” or swiping motion can decrease error rates and improve user satisfaction. Future research is needed to investigate tools and methods to improve the user-friendliness of mobile apps for adults 55 and over, especially those who have little to no experience with touchscreens for whom certain functions may not be completely intuitive.

### Limitations and Lessons Learned

There were some limitations to the current study and some lessons learned. It was a pilot study designed to test a concept. Although ClereMed was designed for use in pharmacies, only 4 participants were actually recruited through a pharmacy. Most recruitment occurred in independent living retirement homes with a research assistant. As a result, our sample population may not be representative of all individuals who pharmacists would assess with ClereMed. Community-dwelling adults who are younger, for example, may differ in their experience with and willingness to adopt new technologies.

On follow-up, pharmacists told us they felt over-burdened recruiting participants and testing the app in the pharmacy setting. For a multi-user app such as ClereMed, the user experience needs to be positive for both experienced and new users. In our design processes, we had input from pharmacists but focused on the patient user and not the pharmacist user. With ClereMed, it is the pharmacist who would make the decision to adopt the app into their practice. The diffusion of innovation model posits that, for a technology to be adopted, it must provide users with a relative advantage over their current circumstances [64]. In hindsight, we should have focused on providing pharmacists with a relative advantage over their current practice. This is an important consideration for future research.

Further, though our goal was to build on the rapid advances in mobile technologies to build a novel tool, another approach would have been to use a simple paper-based tool. The other issue that requires consideration is the motivation of pharmacists to use any tool, be it paper or electronic, to assess and support their patients. Currently, it is likely that many patients who have difficulty reading their prescription labels go unnoticed. More research is needed to explore the ways to work with pharmacists to identify patients who need help. The lack of sensitivity of ClereMed for cognitive impairment also requires further investigation. Nevertheless, if used correctly, an app such as ClereMed has the potential to reduce medication mismanagement in adults 55 and over by rapidly allowing the pharmacist to identify a patient’s inability to read a medication label or understand instructions and provide practical solutions to the problem.

## Abbreviations

HTML: hypertext markup language
IoM: Institute of Medicine
iOS: Apple mobile operating system
ISMP: Institute for Safe Medication Practices
MoCA: Montreal cognitive assessment tool
SUS: systems usability scale
